# Rapid Fluid Velocity Field Prediction in Microfluidic Mixers via Nine Grid Network Model

**DOI:** 10.3390/mi16010005

**Published:** 2024-12-24

**Authors:** Qian Li, Yuwei Chen, Taotao Sun, Junchao Wang

**Affiliations:** Innovation Center for Electronic Design Automation Technology, Hangzhou Dianzi University, Hangzhou 310018, China

**Keywords:** finite element analysis, microfluidic mixer, machine learning

## Abstract

The rapid advancement of artificial intelligence is transforming the computer-aided design of microfluidic chips. As a key component, microfluidic mixers are widely used in bioengineering, chemical experiments, and medical diagnostics due to their efficient mixing capabilities. Traditionally, the simulation of these mixers relies on the finite element method (FEM), which, although effective, presents challenges due to its computational complexity and time-consuming nature. To address this, we propose a nine-grid network (NGN) model theory with a centrally symmetric structure.The NGN uses a symmetric structure similar to a 3 × 3 grid to partition the fluid space to be predicted. Using this theory, we developed and trained an artificial neural network (ANN) to predict the fluid dynamics within microfluidic mixers. This approach significantly reduces the time required for fluid evaluation. In this study, we designed a prototype microfluidic mixer and validated the reliability of our method by comparing it with predictions from traditional FEM software. The results show that our NGN model completes fluid predictions in just 40 s compared to approximately 10 min with FEM, with acceptable error margins. This technology achieves a 15-fold acceleration, greatly reducing the time and cost of microfluidic chip design.

## 1. Introduction

In the past decade, the swift progression of microfluidic technology has resulted in its extensive implementation in various domains, encompassing areas like biotechnology, chemical research, and medical diagnostics [1,2,3]. Microfluidic technology has revolutionized experimental platforms, transitioning from traditional large-scale laboratories to compact, portable chips, often spanning only a few square centimeters or even smaller. These microplatforms can seamlessly integrate multiple independent units, offering features such as lightweight construction, sensitive response, the conservation of expensive reagents, and the efficient utilization of rare samples. As a result, the field of microfluidics has found extensive applications in droplet generation [4], cell sorting [5,6,7], drug screening [8,9,10], organ-on-a-chip technology [11,12,13], single-cell RNA sequencing [14,15], and the prompt detection and management of illnesses like the COVID-19 pandemic [16], and its is also applicable to various other sectors.

Microfluidic devices, specifically designed for mixing, play an essential role in the field of microfluidics. They ensure the efficient and swift blending of various samples at the microscale level [17,18,19]. However, the small size and stringent precision requirements of microfluidic mixers make them vulnerable to even minor deviations, which can significantly impact their performance. Consequently, it is paramount to design an optimally performing microfluidic mixer before manufacturing.

The primary challenge in developing micromixers lies in validating their performance, with experimental validation processes often proving time-consuming and costly. Presently, the FEM stands as a widely used approach to simulate mixer performance. This method involves establishing partial differential equations to model the target system, followed by discretization of these equations to simulate the physical field of the system and obtain numerical solutions. Jain et al. [20] utilized finite element analysis(FEA) to numerically simulate various designs of micromixers and investigated the influence of obstacle channels on performance through experimental research. Similarly, according to the study by Memon et al. [21], they utilized finite element simulation software, such as COMSOL Multiphysics 5.5, to analyze the steady-state and laminar flow characteristics of high Reynolds number viscous fluids through a cylinder fixed between two flat plates. However, despite its effectiveness, this approach presents a high barrier to research and development and entails a lengthy development cycle. Researchers must possess a background in microfluidics and proficiency in specialized FEA software (For example, COMSOL Multiphysics 5.5) to navigate this process effectively.

In recent years, researchers have increasingly turned to computer-aided design and machine learning techniques for the design automation of microfluidic chips [22,23]. One notable approach involves leveraging FEA software to simulate fluid behavior, aiding in the design process prior to chip manufacturing. Typically, this process entails creating the geometric design of the chip and conducting simulations to assess its performance. If the initial design proves unsatisfactory, researchers iterate on the design by returning to the FEA software to refine and predict performance. Ji et al. [24] achieved automated iterative design of stochastic micro mixers through the utilization of ANNs. Similarly, Hong et al. [25] employed machine learning models for the reverse design of microfluidic concentration gradient generators based on topological structures. Their methodology involved generating a dataset comprising 9 million data points derived from a physics-based model, with the required concentration gradient at the outlet serving as the network input and the input parameters and concentrations of the generator serving as the network output. This innovative approach enabled the rapid and efficient design of the desired concentration gradient generator. Furthermore, Wang et al. [26] utilized convolutional neural networks to expedite the prediction of fluid behavior in microfluidic mixers. They represented the geometric shape of an 8 × 8 grid mixer with a 15 × 15 binary digit matrix and generated a training dataset comprising 10,513 random designs. Leveraging convolutional neural networks, they successfully predicted the three outlet velocities and two outlet concentrations of designed micromixers.

However, existing FEA software is often complex, has a steep learning curve, and offers limited customization for microfluidics, making chip simulation challenging for researchers [27]. In our previous work [28] we developed an automated method for designing and optimizing microfluidic mixers. The mixer region was a regular rectangle containing numerous small rectangular obstacles whose specific positions were randomly generated by code. In this study, we adopted a similar method to generate the mixer structures. However, one difference is that we divided the mixer into regular 5 μm × 5 μm grids, and the size of the small obstacles was also set to be one of these grids. This grid partitioning ensures that the computational load remains manageable, as it does not exceed the maximum capacity of our computing devices while also maintaining the prediction accuracy of the trained ANN model.

We propose an NGN model approach based on ANNs for rapidly predicting the fluid field of microfluidic mixers. The mixing area to be predicted is divided into regular grids, and the fluid field can be rapidly predicted under the condition of only boundary values and geometric modeling. This approach utilizes supervised learning in a non-linear regression model, transforming the fluid mechanics problem into a non-linear regression problem that explores the inherent relationships within large datasets. By replacing the complex traditional FEA methods, the ANN approach is approximately 15 times faster in terms of runtime. This method not only overcomes the limitations and complexity associated with traditional FEA software but also provides a more efficient way to predict the fluid behavior of microfluidic mixers. The typical mixing channel width of water-based microfluidic systems is set to 100 μm [29], where the Reynolds number is very low, making turbulent mixing unlikely. Under these conditions, a resolution of 5 μm is sufficient for finite element simulation of flow velocity without compromising prediction accuracy. This iterative prediction process continues until the velocities at all positions in the target system are predicted, demonstrating the efficiency and effectiveness of our proposed NGN model approach.

## 2. Theory of Predicting Microfluidic Mixer Velocity

### 2.1. The NGN Model

**Figure 1:**
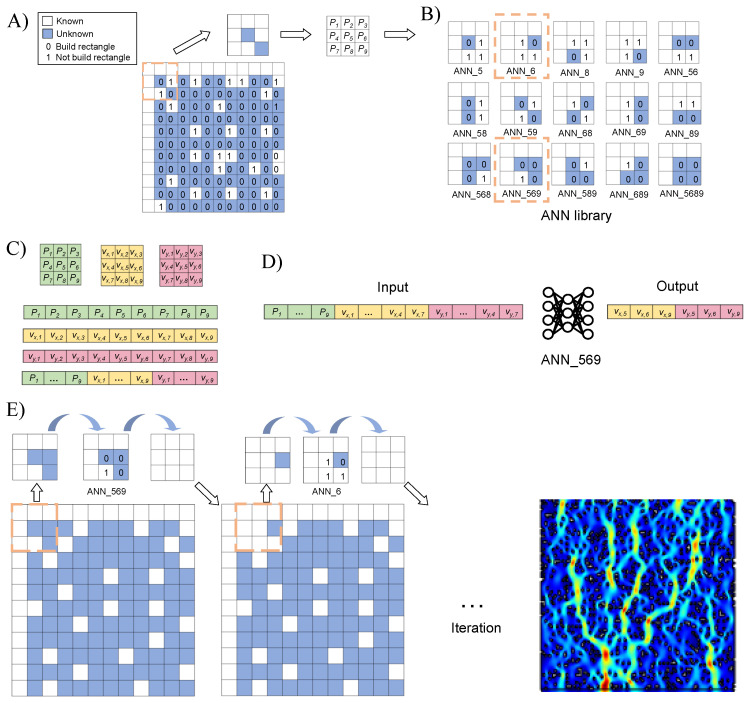
Introduction and usage example of nine-grid network model. (**A**) Meshing of microfluidic mixer. (**B**) ANN libarary. (**C**) Dataset structure. (**D**) Example input and output for the ANN_569 model. (**E**) An example of predicting the fluid field of the target system using the nine-gride network model.

By modularizing the fluid field grid of the micromixer and under known information and boundary conditions, block-wise iterative prediction is performed on the unbounded matrix. This process obtains the dataset structure for the ANN model. As shown in Figure 1A, the mixing area of the micromixer is divided into a grid matrix. Each grid can exist in two states: a built matrix (“1” represents built) and an unbuilt matrix (“0” represents unbuilt). It is defined that the built matrix has a velocity of 0, and the unbuilt matrix has a combined velocity of $v_{x}$ and $v_{y}$. The blue areas indicate grids with known information, while the white areas represent grids with unknown information. The grid velocities in the built matrix are initialized to 0, while the grid velocities in the unbuilt matrix ($v_{x}$, $v_{y}$) are initialized to −1. Three matrices of the same size are used to represent the geometry of the mixer, $v_{x}$, and $v_{y}$.

To solve for the mixing region in the microfluidic mixer while adding boundary conditions, the first row and first column of the grid in Figure 1A represent known boundary conditions. The velocities of the upper and left boundaries of the target mixer are appended to the first row and first column positions of the $v_{x}$ and $v_{y}$ matrices. Positions with a velocity of 0 are considered as built rectangles and are mapped to “1” in the geometry structure matrix, while positions with non-zero velocities are considered as unbuilt rectangles and are mapped to “0” in the geometry structure matrix.

A nine-grid structure is defined, with cells labeled P1 through P9 from left to right and top to bottom, as shown in Figure 1B. The first nine-grid cell’s P1, P2, P3, P4, and P7 are known boundary conditions, and they are based on whether rectangles are built or not, meaning that 15 possible scenarios are derived: {0111, 1011, 1101, 1110, 0011, 0101, 0110, 1001, 1010, 1100, 0001, 0010, 0100, 1000, 0000}. Each of these corresponds to an ANN model named {ANN_5, ANN_6, ANN_8, ANN_9, ANN_56, ANN_58, ANN_59, ANN_68, ANN_69, ANN_89, ANN_568, ANN_569, ANN_589, ANN_689, ANN_5689}. Figure 1C describes the dataset structure of the ANN models. Slices of the geometry structure matrix, $v_{x}$, and $v_{y}$ are taken using the nine-grid structure. This results in three nine-grid slices (green is the geometry structure matrix slice, yellow is the $v_{x}$ matrix slice, and pink is the $v_{y}$ matrix slice). These slices are then flattened into one-dimensional arrays, which are concatenated to form a dataset array with 1 row and 27 columns. To accurately simulate the complex flow field within microfluidic mixers, we have trained an ANN model. Specifically, we have selected 15 different meticulously trained ANN models from a library of ANNs that have been tailored to our dataset of data arrays within a nine-grid range. Upon completion of this process, we redefined new nine-grid structures and iteratively employed these models to predict velocity vectors across the entire mixer space, thereby achieving comprehensive flow field prediction.

After training the ANN model, we performed predictions of the velocity vector fields within the flow field of the mixer. Given the known boundary conditions, to forecast the intricate flow field within the microfluidic mixer, we trained 15 distinct ANN models. For any dataset array within the nine-grid range, the database contains an appropriate ANN model for predicting the velocity vectors. After predicting the velocity vectors for the initial nine-grid range, a new nine-grid area is identified in subsequent iterations to complete the predictions throughout the entire mixer space.

Taking the target system shown in Figure 1E as an example, this section describes the process of using the NGN model method to predict the flow velocities within a microfluidic mixer system. Starting from the top left corner of the target system, the first step is to isolate the first nine-grid cells (outlined by the orange dashed box) to form an NGN for prediction. In this NGN, the predicted parameters for P5, P6, P8, and P9 correspond to values of 0, 0, 1, and 0, respectively, so the ANN model numbered ANN_569 is needed for prediction in this area. The corresponding ANN model is then used to predict the velocities at these positions (the input–output data structure is shown in Figure 1D). The predicted results are then updated in the target system, completing the prediction for the first NGN unit.

Next, by shifting one grid cell horizontally, the second NGN is isolated for another prediction. In the second NGN, the predicted parameters for P5, P6, P8, and P9 correspond to values of 1, 0, 1, and 1, respectively, so the ANN model numbered ANN_6 is required for this area. The corresponding ANN model is called to predict the flow velocities at the specified positions, and the results are updated in the target system, completing the prediction for the second nine-grid unit. This iterative prediction process continues until the velocities at all positions in the target system are predicted.

### 2.2. Building a Dataset Using FEA

To validate the practical performance of the proposed NGN model method based on ANNs, a classic microfluidic mixer was designed, and its fluid field was predicted. The 15 types of ANNs summarized in this section used supervised learning machine learning models to predict fluid velocities based on the geometric structures and boundary conditions. Therefore, a database of fluid field distributions for microfluidic mixers with different geometric designs was established. This database served as the dataset for subsequent training.

**Figure 2:**
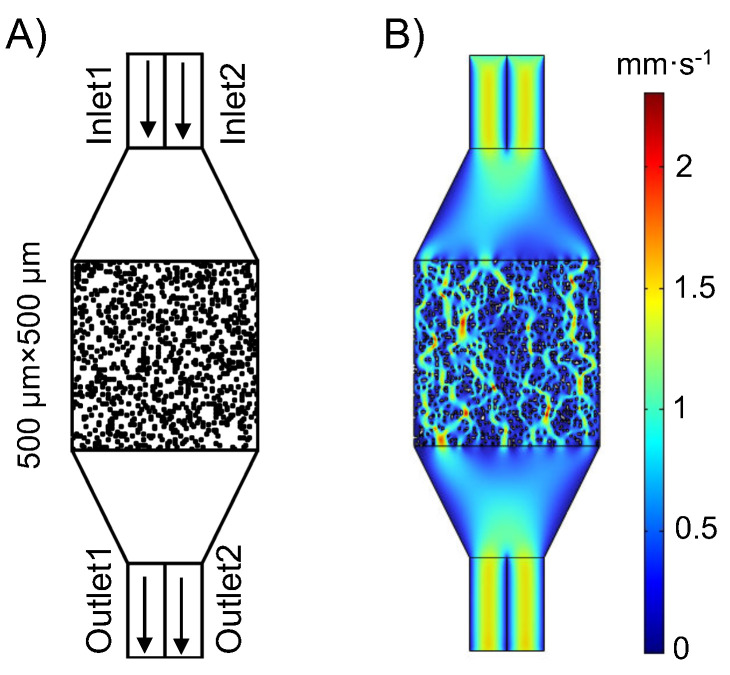
(**A**) The geometric structure diagram of a microfluidic mixer, which has two inlets, two outlets, and a 500 μm × 500 μm reaction zone. (**B**) An example shows the fluid velocity field predicted by the FEM of a randomly generated mixer.

The schematic of the geometry structure of this micromixer is shown in Figure 2A. It consists of two inlets (each with a size of 100 μm × 250 μm), two outlets (each measuring 100 μm × 250 μm), and a mixing region measuring 500 μm × 500 μm. The distribution of the fluid field is shown in Figure 2B. In constructing the three essential data matrices that encapsulate the mixer’s intricacies, our core emphasis lies in meticulously analyzing and precisely quantifying both the structural layout and fluid dynamics within its confined interior. To uphold the experiment’s reliability, we meticulously evaluated key parameters, including the grid’s fineness within the nine-grid partition, the mixer’s precise geometric dimensions, the fluid’s velocity vectors, and the crucial boundary conditions that govern the mixer’s operation, such as inlet velocity, outlet pressure, and wall conditions. By judiciously selecting and quantifying these parameters, we assembled three highly accurate and dependable $v_{x}$, $v_{y}$, and geometry data matrices, which provided support for subsequent design enhancements and optimization endeavors.

**Figure 3:**
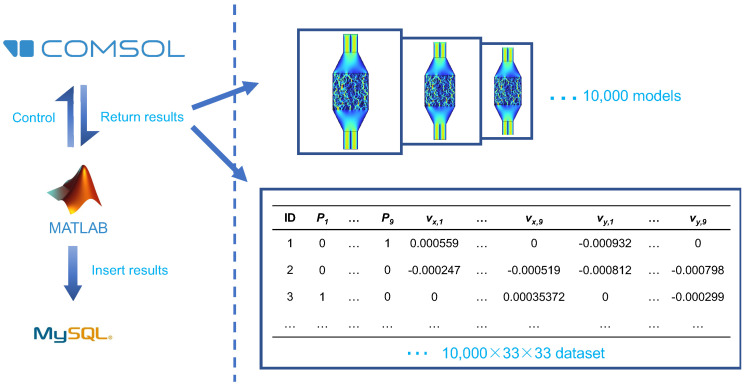
The design process of a random microfluidic mixer. Within a month, 10,000 random microfluidic mixer chip designs were generated using MATLAB R2020b programs, and the performance of each chip was simulated using COMSOL 5.5. Finally, the results were saved in a MySQL 5.7.16 database.

During the simulation, we incorporated two coupled physical domains—Laminar Flow and Transport of Diluted Species (TDS)—with the channel filled with incompressible water. In the COMSOL Multiphysics 5.5 software, the constant velocity at each inlet of the laminar flow field was set to 0.001 m/s, while the outlet boundary condition was specified as 0 Pa pressure. The channel walls were defined as no-slip boundaries, indicating no relative motion between the water flow and the walls. For the transport of diluted species, Inlet 1 was designated with an inflow concentration of 0.001 mol/m^3^, while the boundary condition at Inlet 2 was set to 0 mol/m^3^. Both outlets were defined as outflow boundaries, and we utilized a diffusion coefficient determined to be 4.25× 10^−10^ m^2^s^−1^. Grid independence studies were conducted as needed, and the model was meshed with over 70,000 grid nodes. Two separate solvers were set up to solve for laminar flow and dilute species transport. The fluid field distribution in the mixer is as shown in Figure 2B. To simulate a variety of different chip fluid fields, the mixing region was divided into a 100×100 grid, with each grid having a resolution of 5 μm × 5 μm. This allowed for representing different mixer designs using random 100×100 matrices. The mixer simulations were implemented using FEA software COMSOL Multiphysics 5.5, and MATLAB API provided by COMSOL was used to automate the performance simulation of chips. The resulting fluid field distribution is presented in Figure 3.

The data generated from these simulations using the concept of nine-grid slicing for the geometry structure matrix, as well as $v_{x}$ and $v_{y}$ matrices with a step size of 3 × 3, resulted in collecting 33 × 33 data points for each model. In total, 10,000 different mixer designs were randomly generated. Finally, the constructed dataset and simulation models were stored in a local MySQL database.The data generated from these simulations using the concept of nine-grid slicing for the geometry structure matrix, as well as $v_{x}$ and $v_{y}$ matrices with a step size of 3 × 3, resulted in collecting 33 × 33 data points for each model. In total, 10,000 different mixer designs were randomly generated. Finally, the constructed dataset and simulation models were stored in a local MySQL database.

In the testing environment, we focused on internal mixing zone changes, keeping inlet external structure and fluid boundary conditions constant. Random obstacles in the zone significantly affect fluid velocity. While external changes may influence mixing, internal structural variations have a greater impact on velocity and mixing outcomes. Import flow rates alter internal flow to some extent. Thus, we strictly controlled the input parameters and boundary conditions within the model prediction ranges to prevent overfitting.

### 2.3. Training ANN Models to Predict the Fluid Field of a Microfluidic Mixer

Based on the PyTorch framework, the training process was implemented in Python 3.6, with 2000 randomly selected data sets from 10,000 randomly designed microfluidic mixer models. The total dataset size was $2,000^{33\times 33}$. Following the data cleansing phase, a stratified random sampling technique was employed to allocate 80% of the dataset to the training phase, with the remaining 20% earmarked for validation purposes. Throughout the neural network’s training regimen, the Mean Absolute Error metric was leveraged to assess the performance by quantifying the discrepancy between predicted and actual outcomes. The Adam optimizer was chosen for training. The accuracy of $v_{x}$ was calculated using Equation (1), and the accuracy of $v_{y}$ was calculated using Equation (2). For the training process of the ANN and simulation of the micromixer, we chose to run it on a high-performance server installed with a Linux operating system. The server is equipped with RTX 2070 GPU graphics card and 256 GB RAM. However, our study revealed that for everyday applications involving fluid prediction utilizing nine-lattice network models, a general-purpose lightweight office laptop is more than adequate, offering a practical and efficient solution.

(1) $$xflowANN_{accuracy}=1-\frac{1}{n}\sum_{k=1}^{n}\left(\frac{|\Delta v_{(x,out),k}|}{v_{(x,out),k}}\right)$$

In Equation (1), *k* indicates the index of individual features within the dataset used for training or testing, while *n* denotes the total count of iterations within the training or testing process. $\Delta v_{(x,out),k}$ represents the difference between the predicted horizontal component velocity $v_{x}$ of the fluid predicted by the ANN and the target value, which is the target value of the fluid velocity.

(2) $$yflowANN_{accuracy}=1-\frac{1}{n}\sum_{k=1}^{n}\left(\frac{|\Delta v_{(y,out),k}|}{v_{(y,out),k}}\right)$$

In Equation (2), $\Delta v_{(y,out),k}$ represents the difference between the vertical component velocity $v_{y}$ predicted by the ANN and the target value, which is the target value of fluid velocity.

The equation is used to calculate the accuracy of $v_{y}$, measuring how closely the ANN’s predictions align with the target values for fluid velocity. This accuracy calculation helps assess how well the model is performing in predicting the vertical fluid velocity component.

After thorough experimentation and analysis, we selected an ANN model with a fully connected seven-layer structure, with each layer containing 400 neuron nodes, and Leaky ReLU as the key activation function. During the training process, three parameters were monitored for each iteration: the training set accuracy, test set accuracy, and training set error. A successful training process typically involves the training error gradually converging from high to low and both the training set accuracy and test set accuracy steadily increasing. The test set accuracy is often lower than the training set accuracy and ultimately stabilizes.

This process of parameter tuning and training is critical to achieving the desired performance of the ANNs for predicting fluid behavior in microfluidic mixers. It helps ensure that the models are learning effectively and generalizing well to unseen data, as evidenced by the convergence of training errors and the increase in accuracy. In the experiment, the ANN model achieved an outstanding training accuracy of 99.8%, while the nine-grid model method also achieved remarkable progress in enhancing efficiency. Specifically, compared to the traditional FEM, the nine-grid model method shortened the prediction time for a classic microfluidic mixer by 15 times, requiring only 40 s. Moreover, if this method is deployed on a server equipped with GPU chips and leverages the powerful parallel computing capabilities of the GPU, the overall computation time is expected to be further reduced, ushering in a new era of more efficient and real-time predictive analysis. In the context of pursuing low costs and high-speed predictions, this method not only maintains reasonable prediction accuracy but also optimizes computational resources and time costs to the utmost extent, undoubtedly providing a practical solution for related fields.

## 3. Results and Discussion

### 3.1. Training of the ANN Library

**Figure 4:**
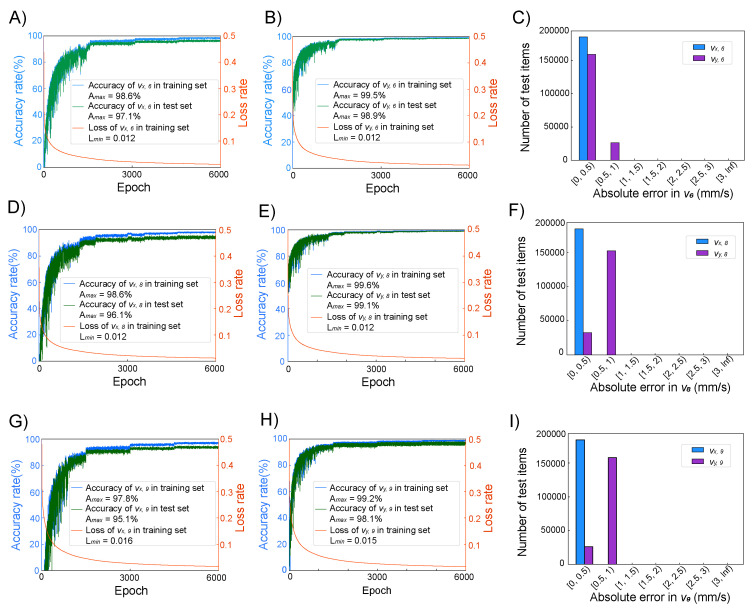
(**A**) The training curve of the $v_{x,6}$ of ANN_6 during 6000 epochs. (**B**) The training curve of the $v_{y,6}$ of ANN_6 during 6000 epochs. (**C**) The histogram of the absolute error of $v_{x,6}$ and $v_{y,6}$ of 185,337 items in the test set. (**D**) The training curve of the $v_{x,8}$ of ANN_8 during 6000 epochs. (**E**) The training curve of the $v_{y,8}$ of ANN_8 during 6000 epochs. (**F**) The histogram of the absolute error of $v_{x,8}$ and $v_{y,8}$ of 185,337 items in the test set. (**G**) The training curve of the $v_{x,9}$ of ANN_9 during 6000 epochs. (**H**) The training curve of the $v_{y,9}$ of ANN_9 during 6000 epochs. (**I**) The histogram of the absolute error of $v_{x,9}$ and $v_{y,9}$ of 185,337 items in the test set.

From Figure 4A, it can be observed that after 6000 iterations of training, the training dataset achieved an accuracy of 98.6% for $v_{x,6}$, while the testing dataset recorded an accuracy of 97.1%. After the same number of iterations, Figure 4B indicates that the accuracy for $v_{y,6}$ in the training dataset came out to 99.5%, and the testing dataset achieved an accuracy of 98.9%. The ANN_6 model demonstrated a loss rate that eventually stabilized at 0.012.

In Figure 4C, the absolute error within the test dataset, which is composed of 185,337 data points, is displayed. For $v_{x,6}$, all absolute errors were less than 0.5 mm/s, and 85.9% of the absolute errors for $v_{y,6}$ were also less than 0.5 mm/s. When considering an absolute error threshold of 1 mm/s, the percentage of $v_{y,6}$ with errors below this threshold quickly increased to 99.9%. This indicates that the predictions for $v_{x,6}$ and $v_{y,6}$ made by the ANN_6 model are highly consistent with the predictions from COMSOL, demonstrating a high degree of accuracy.

Figure 4D shows that after 6000 iterations of training, the ANN_8 model for $v_{x,8}$ had a training set accuracy of 98.6% and a test set accuracy of 96.1%. Figure 4E indicates that for $v_{y,8}$, after 6000 iterations of training, the training set accuracy came out to 99.6%, and the test set accuracy came out to 99.1%. Figure 4F presents the absolute errors in the test set: for $v_{x,8}$, 17.5% of the data had errors less than 0.5 mm/s, while 82.5% of the data had errors between 0.5 mm/s and 1 mm/s. This suggests that the predictions made by the ANN_8 model are also highly consistent with the results predicted by COMSOL.

**Figure 5:**
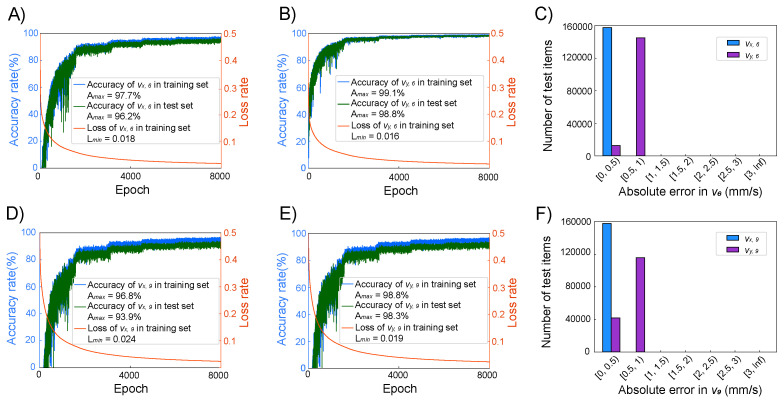
(**A**) The training curve of the $v_{x,6}$ of ANN_69 during 8000 epochs. (**B**) The training curve of the $v_{y,6}$ of ANN_69 during 8000 epochs. (**C**) The histogram of the absolute error of $v_{x,6}$ and $v_{y,6}$ of 157,763 items in the test set. (**D**) The training curve of the $v_{x,9}$ of ANN_69 during 8000 epochs. (**E**) The training curve of the $v_{y,9}$ of ANN_69 during 8000 epochs. (**F**) The histogram of the absolute error of $v_{x,9}$ and $v_{y,9}$ of 157,763 items in the test set.

In Figure 5A, it can be seen that after 8000 iterations of training, the training set accuracy for $v_{x,6}$ was 97.7%, and the test set accuracy was 96.2%. Figure 5B shows that for $v_{y,6}$, after 8000 iterations of training, the training set accuracy was 99.1%, and the test set accuracy was 98.8%. In Figure 5D, it is observed that after 8000 iterations of training, the training set accuracy for $v_{x,9}$ was 96.8%, and the test set accuracy was 93.9%. Figure 5E indicates that for $v_{y,9}$, after 8000 iterations of training, the training set accuracy was 98.8%, and the test set accuracy was 98.3%. Figure 5C,F describe the absolute errors in the test set for the velocities at positions P6 and P9, all of which had errors less than 1 mm/s.

**Figure 6:**
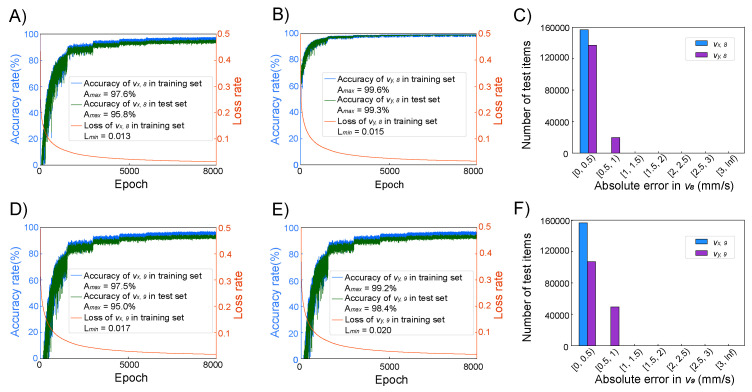
(**A**) The training curve of the $v_{x,8}$ of ANN_89 during 8000 epochs. (**B**) The training curve of the $v_{y,8}$ of ANN_89 during 8000 epochs. (**C**) The histogram of the absolute error of $v_{x,8}$ and $v_{y,8}$ of 156,774 items in the test set. (**D**) The training curve of the $v_{x,9}$ of ANN_89 during 8000 epochs. (**E**) The training curve of the $v_{y,9}$ of ANN_89 during 8000 epochs. (**F**) The histogram of the absolute error of $v_{x,9}$ and $v_{y,9}$ of 156,774 items in the test set.

In Figure 6A, it can be seen that after 10,000 iterations of training, the training set accuracy for $v_{x,6}$ was 97.6%, and the test set accuracy was 95.8%. Figure 6B shows that for $v_{y,8}$, after 10,000 iterations of training, the training set accuracy was 99.6%, and the test set accuracy was 99.3%. In Figure 6D, it is observed that the training set accuracy for $v_{x,9}$ was 96.8%, and the test set accuracy was 93.9%. Figure 6E indicates that for $v_{y,9}$, after 8000 iterations of training, the training set accuracy was 98.8%, and the test set accuracy was 98.3%. Figure 6C,F describe the absolute errors in the test set for the velocities at positions P8 and P9, all of which had errors less than 1 mm/s.

Compared to ANN_6, ANN_8, and ANN_9, ANN_69 and ANN_89 showed more significant fluctuations in accuracy. This is because the dataset contains a total of 27 features as inputs and outputs. When the neural network has more outputs, it receives less information from the nine-grid, leading to more significant fluctuations in weight updates during training and increased difficulty in predictions.

The training process of the ANN models was similar, and for brevity, it will not be discussed here in details. The training results are presented in Table 1. However, it is worth noting that as the number of outputs predicted by ANN models increased, the training accuracy of the neural network was slightly lower. This is because the total amount of information that the NGN model can provide is constant (with a maximum of 25 inputs and 2 outputs and a minimum of 19 inputs and 8 outputs). The more positions you predict, the less information the model can use as input, which may result in a slightly lower neural network performance. In the design of microfluidic mixers, the sensitivity of model prediction accuracy to variations in input parameters and boundary conditions is paramount. Unreasonable parameters or boundary conditions can lead to significant discrepancies between predictions and actual performance, thereby affecting the practicality and effectiveness of the design. Therefore, during the design process, we strictly controlled the rationality of input parameters and boundary conditions to ensure they would fall within the valid range of the model predictions. This prevents overfitting and safeguards the accuracy and reliability of the design.

**Figure 7:**
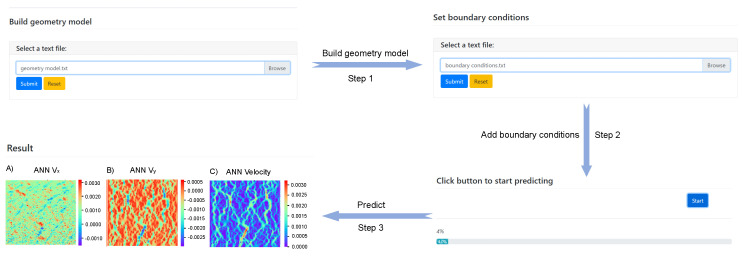
The step of predicting fluid velocity field of the microfluidic chip using ANN Tool.

After training 15 ANN models, we successfully predicted the flow field in microfluidic chip design without utilizing FEA and relying solely on the geometric model and boundary conditions. To facilitate the prediction process, we developed a user-friendly graphical user interface (GUI) tool called the ANN Tool, which visualizes the ANN prediction process. Predicting the flow field in microfluidic chips involves only three simple steps, as illustrated in Figure 7.

The user uploads the geometric model file of the microfluidic chip and the boundary conditions file. Then they click the “Start” button, and the prediction process begins, with real-time progress displayed. Upon completion, the ANN Tool displays images of the x-direction flow field, y-direction flow field, and the total flow field on the web page. Using our ANN library to predict the velocity of the flow field in microfluidic chips is approximately 15 times faster than previous FEM techniques.

### 3.2. Quantitative Analysis of the ANN Library

**Figure 8:**
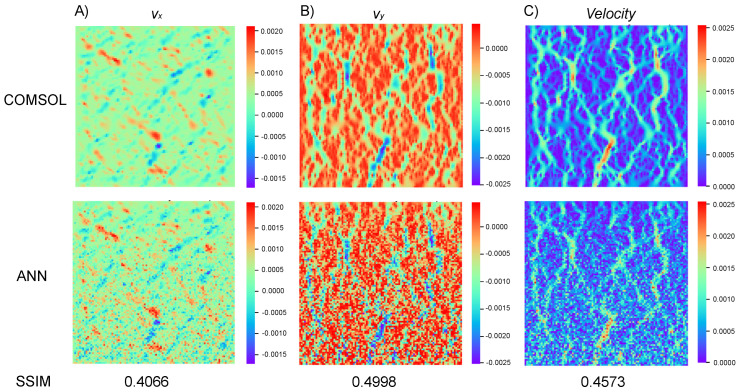
The velocity distribution in the reaction zone was predicted by COMSOL and ANN methods. The corresponding SSIM values between the two methods are listed for quantitative analysis.

To further validate the performance of the nine-grid ANN model, 500 microfluidic mixer flow field simulations, as shown in Figure 8, were randomly designed and simulated using the FEA method. A Python program was written to automatically call the pre-trained ANN models from the library based on the 01 matrix representing the geometric model of the microfluidic mixer in order to predict the entire flow field of the mixer. Finally, the Structural Similarity Index (SSIM) algorithm was used to calculate the similarity between the flow field images obtained from finite element simulations and those predicted using the ANN method.

Figure 8 shows three parts, with the first part presenting the flow field predicted by the COMSOL simulation software, including the horizontal component $v_{x}$, vertical component $v_{y}$, and overall fluid field velocity. The second part horizontally represents the flow field predicted by the nine-grid ANN model (horizontal component, vertical component, and overall fluid field). The SSIM algorithm was used to compare the similarity of the two images in Figure 8A, resulting in a numerical value of 0.4066. Similarly, for Figure 8B, a value of 0.4998 was obtained, and for Figure 8C, a value of 0.4573 was obtained. As can be seen in the figure, the errors have accumulated due to the constant calling of the ANN model, which led to more inaccurate data the further away from the inlet. However, in the mixing region, the trend of the ANN model predicting the flow velocity was still consistent with the data from the COMSOL simulation.

**Figure 9:**
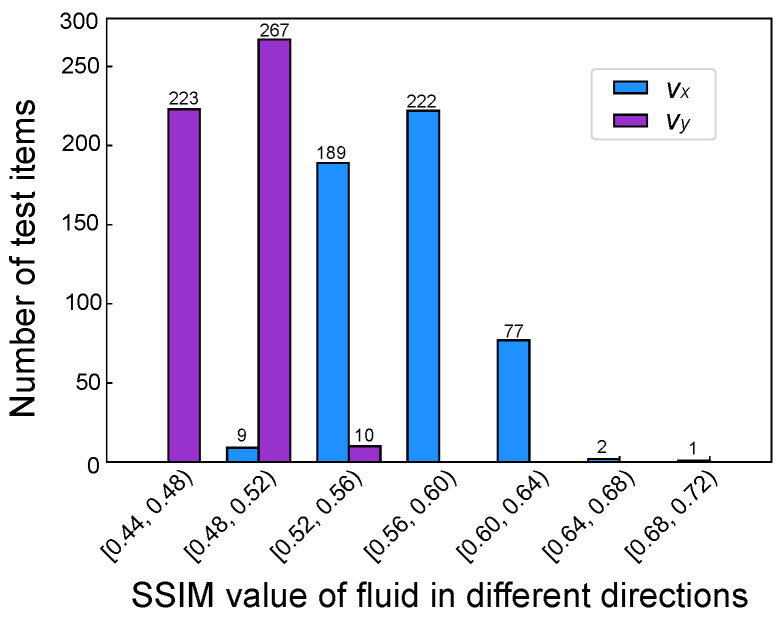
The COMSOL and ANN methods were used to predict the velocity distribution of 500 new microfluidic chip reaction zones, and the distribution of the corresponding SSIM values between the two methods was calculated.

The results shown in Figure 9 indicate the SSIM values for the randomly generated 500 microfluidic mixer designs. In blue, the SSIM values for the horizontal component of the flow field are shown. A small number of designs have SSIM values ranging from 0.68 to 0.72, while 99.4% of the designs have SSIM values between 0.48 and 0.64. In purple, the SSIM values for the vertical component of the flow field are presented. Some designs have SSIM values between 0.52 and 0.56, and 98% of the designs have SSIM values between 0.44 and 0.52.

This demonstrates the successful prediction of the microfluidic mixer’s flow field in the mixing domain using the nine-grid ANN model. Although our developed method did not achieve one hundred percent accuracy in prediction, it achieved remarkable results in significantly enhancing efficiency. Specifically, the overall computation time was drastically reduced from the original 10 min to less than 40 s, yielding an acceleration of at least 15 times. This substantial decrease significantly alleviates the pressure on computational resources and shortens the time cost. Importantly, this achievement was made while maintaining a certain level of reliability in the prediction results, which is crucial for prediction applications that prioritize both efficiency and cost-effectiveness. Moreover, if this method is deployed on servers equipped with GPU chips, leveraging the powerful parallel computing capabilities of GPUs, the overall computation time has the potential to be further shortened, ushering in a new era of more efficient and real-time predictive analysis. In the context of pursuing both low cost and high-speed prediction, our method undoubtedly offers a practical solution in relevant fields, maximizing the optimization of computational resources and time cost while maintaining a reasonable range of prediction accuracy.

The study found that the NGN model method is limited by the dimension of grid division. For instance, the mixer designed in this chapter was divided into a 100×100 grid with a precision of 5 μm × 5 μm, theoretically allowing for $2^{100\times 100}$ mixer designs, yet it is clearly impractical to simulate all of them. In practical applications, striking a balance between the grid division size and precision of the mixer is crucial to achieving optimal performance. Our future research will focus on further refining the existing network structure to enhance its applicability in real-world microfluidic applications.

### 3.3. Three Designs for the Proof of Concept

**Figure 10:**
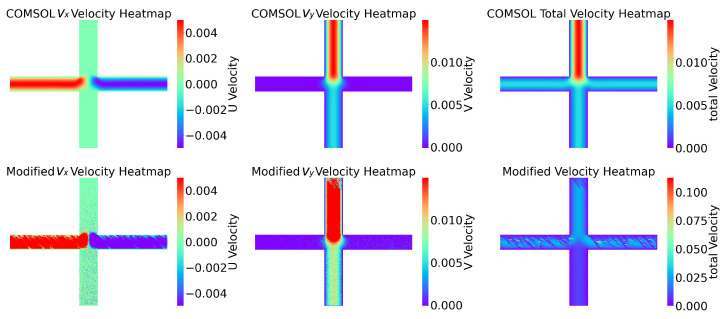
The SSIM for $v_{x}$ is 0.7646, the SSIM for $v_{y}$ is 0.7597, and the SSIM for the total velocity is 0.6459.

**Figure 11:**
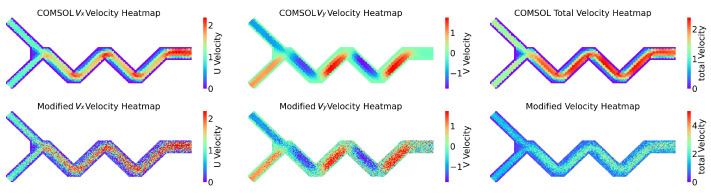
The SSIM for $v_{x}$ is 0.5494, the SSIM for $v_{y}$ is 0.5489, and the SSIM for the total velocity is 0.5507.

**Figure 12:**
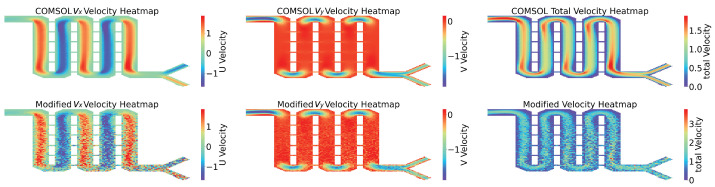
The SSIM for $v_{x}$ is 0.5497, the SSIM for $v_{y}$ is 0.5496, and the SSIM for the total velocity is 0.5227.

Clearly, the NGN model’s predictive prowess varies across fluids in different mixer configurations due to their unique characteristics. To enhance the experiment’s testing scenarios and further validate the effectiveness and reliability of the NGN model’s prediction method, we developed three types of mixers (cross mixer, mixer 1, and mixer 2) for conceptual proof, with each displaying unique features in mixing efficiency and fluid resistance. If the NGN model can accurately predict spatial velocity vectors for these three mixers, it implies that the prediction is not significantly influenced by the mixer type, thus demonstrating the model’s universal applicability in predicting mixer fluid dynamics. Post-simulation, the results are illustrated in Figure 10, Figure 11, and Figure 12, with SSIM scores of 0.6459, 0.5507, and 0.5227, respectively. The fluid prediction outcomes for all three mixers are relatively accurate, clearly showing the NGN model’s effectiveness and reliability in predicting fluid behavior across various mixers.

### 3.4. Potential Applications of the Proposed NGN Model

#### 3.4.1. Microfluidic Mixer for Sample Preparation

Microfluidic mixers are critical for precise and efficient sample preparation in bioengineering and chemical experiments. In applications such as clinical diagnostics, pharmaceutical development, and point-of-care testing, accurate preparation of reagent-sample mixtures ensures reliable analytical results [30].

The proposed NGN-based ANN model is able to accelerate the prediction of fluid dynamics, making it feasible to optimize mixer designs iteratively in real time. This can improve the performance of microfluidic mixers used to homogenize biological samples (e.g., blood, saliva) with reagents or buffers at micro- and nano-liter scales. Faster computational evaluations enable the design of mixers that achieve uniform concentration gradients in shorter timeframes, which is especially critical for time-sensitive assays like PCR, immunoassays, or nucleic acid testing [31]. By reducing design time and cost, the proposed method allows researchers to adapt mixers for various biomolecular applications without extensive reliance on computationally expensive FEM simulations.

#### 3.4.2. Bioreactors to Perform Chemical Reactions in Micro-/Nano-Liter Volumes

Microfluidic bioreactors are increasingly used to perform highly controlled chemical and biological reactions in micro- and nano-liter volumes [32,33]. These platforms enable precise control over reaction parameters such as flow rate, temperature, and reactant mixing, which are vital for applications like enzyme kinetics studies [34], cell culture [35], and drug screening [36].

The NGN-based ANN model enhances the design process of microfluidic bioreactors by enabling rapid predictions of fluid behavior and mixing efficiency. Accurate velocity profiles and flow predictions allow optimization of conditions to achieve efficient reagent distribution, reduce dead zones, and enhance reaction yields. In particular, for reactions sensitive to mixing time and concentration gradients, such as protein crystallization or enzymatic reactions, the rapid design and evaluation facilitated by our method can lead to improved reactor performance.

#### 3.4.3. Microcooling Applications for Heterogeneous Integration-Based IC/Microsystems

With the increasing miniaturization and integration of microelectronics, efficient heat dissipation has become a critical challenge for maintaining system performance and reliability [37]. Microfluidic cooling systems, particularly those integrated with heterogeneous ICs and microsystems, offer highly localized and efficient thermal management.

The proposed NGN model enables rapid evaluation of fluid flow within microfluidic cooling channels, aiding in the design of optimized mixer geometries for effective heat transfer [38]. By predicting the velocity profile, our method can facilitate designs that enhance convective heat transfer within microscale channels. This is particularly useful for cooling high-power-density components, such as 3D-stacked chips or integrated photonic–electronic systems [39].

Compared to FEM-based simulations, the significantly reduced computational time allows for more iterations in optimizing fluidic cooling geometries, fluid types, and flow conditions. This acceleration translates into faster prototyping and deployment of microcooling solutions for emerging heterogeneous integration technologies.

## 4. Conclusions

This research introduces a NGN model combined with ANNs for the accelerated prediction of fluid fields in microfluidic mixers. The mixer is discretized into 5 μm × 5 μm cells grouped into 3×3 grid blocks. Matrices $v_{x}$, $v_{y}$, and geometry describe the flow field and geometry post-division. Under known boundary conditions, the first nine-grid block yields 15 unique outcomes, as shown in Figure 1B. During the prediction process, a carefully trained set of 15 ANN models from the ANN library are utilized to predict the first nine vector arrays of the grid-range dataset. After predicting a nine-grid block, we redefine a new block and repeat until entire mixer’s fluidic behavior is predicted. To validate the effectiveness of this approach, a classical microfluidic mixer was designed, and its mixing domain was meshed. The proposed nine-grid method was used for the swift prediction of the flow field within the mixing domain. Using the COMSOL finite element simulation software to simulate the mixer, it was found that the NGN model method predicted the mixer’s flow field in less than 40 s, while traditional FEA methods took 10 min. By combining the low-cost NGN model with ANN models, an exceptionally economical and efficient prediction method for fluid behavior has been achieved. Compared to the more complex FEM, the NGN model boasts lower hardware requirements and simpler operation, significantly reducing the consumption of computer resources and time costs.

Microfluidic mixers of any shape can theoretically be meshed and predicted fluid field using the proposed NGN model method, this could reduce the design threshold for microfluidic mixers and expand their applications. The integration of the low-cost NGN model and ANN models can accelerate flow velocity predictions, although errors increase with the number of iterations.

When traditional FEA methods solve flow fields, the solver for the target system first iterates multiple times based on boundary conditions to obtain an optimal solution, which is then used as known conditions for solving the next set of partial differential equations. This iterative process is often both complex and time-consuming. The proposed NGN-based ANN model overcomes traditional FEM limitations by drastically reducing computation time while maintaining accuracy. It benefits microfluidic mixer designs, enhancing sample preparation, bioreactor development, and microcooling systems. This accelerates innovation and cuts costs in biomedical diagnostics, chemical synthesis, and electronics cooling, promoting wider adoption of microfluidic technologies in research and industry.

In the future, the NGN model method can be applied to provide initial solutions for FEA methods, reducing the number of iterations and improving their computational speed. Therefore, exploring the fluid performance and automating the optimization of microfluidic chips based on artificial intelligence technology holds valuable and practical significance.

## Figures and Tables

**Table 1:** Other ANN models training results.

ANN	Position	Training Accuracy	Testing Accuracy	Training Loss	ANN	Position	Training Accuracy	Testing Accuracy	Training Loss
ANN_5	$v_{x,5}$	97.5%	96.8%	0.019	ANN_568	$v_{x,5}$	97.9%	97.5%	0.015
	$v_{y,5}$	99.0%	98.6%	0.020		$v_{y,5}$	99.5%	99.4%	0.017
ANN_6	$v_{x,6}$	98.6%	97.1%	0.012		$v_{x,6}$	96.8%	96.6%	0.017
	$v_{y,6}$	99.5%	98.9%	0.012		$v_{y,6}$	99.2%	99.1%	0.020
ANN_8	$v_{x,8}$	98.6%	96.1%	0.012		$v_{x,8}$	96.9%	96.6%	0.019
	$v_{y,8}$	99.6%	99.1%	0.012		$v_{y,8}$	99.1%	99.0%	0.016
ANN_9	$v_{x,9}$	97.8%	95.1%	0.016	ANN_569	$v_{x,5}$	96.8%	96.7%	0.014
	$v_{y,9}$	99.2%	98.1%	0.015		$v_{y,5}$	99.4%	99.3%	0.018
ANN_56	$v_{x,5}$	98.6%	97.9%	0.012		$v_{x,6}$	96.9%	96.7%	0.020
	$v_{y,5}$	99.6%	99.5%	0.015		$v_{y,6}$	99.2%	99.0%	0.020
	$v_{x,6}$	98.0%	97.2%	0.015		$v_{x,9}$	95.4%	93.8%	0.027
	$v_{y,6}$	99.5%	99.2%	0.016		$v_{y,9}$	98.5%	98.3%	0.023
ANN_58	$v_{x,5}$	98.7%	98.0%	0.012	ANN_589	$v_{x,5}$	97.0%	96.8%	0.015
	$v_{y,5}$	99.6%	99.4%	0.012		$v_{y,5}$	98.9%	98.8%	0.015
	$v_{x,8}$	98.1%	96.6%	0.015		$v_{x,8}$	96.6%	96.1%	0.019
	$v_{y,8}$	99.5%	99.2%	0.014		$v_{y,8}$	99.3%	99.1%	0.020
ANN_59	$v_{x,5}$	98.4%	97.7%	0.012		$v_{x,9}$	95.6%	94.7%	0.023
	$v_{y,5}$	99.5%	99.4%	0.014		$v_{y,9}$	98.9%	98.5%	0.027
	$v_{x,9}$	97.2%	95.1%	0.020	ANN_689	$v_{x,6}$	96.4%	95.7%	0.021
	$v_{y,9}$	99.1%	98.4%	0.019		$v_{y,6}$	98.6%	98.5%	0.020
ANN_68	$v_{x,6}$	98.2%	97.2%	0.015		$v_{x,8}$	95.5%	94.3%	0.021
	$v_{y,6}$	99.4%	99.0%	0.015		$v_{y,8}$	99.2%	99.1%	0.022
	$v_{x,8}$	97.8%	96.5%	0.015		$v_{x,9}$	95.5%	94.2%	0.029
	$v_{y,8}$	99.5%	99.2%	0.015		$v_{y,9}$	98.8%	98.4%	0.029
ANN_69	$v_{x,6}$	97.7%	96.2%	0.018	ANN_5689	$v_{x,5}$	93.2%	92.2%	0.030
	$v_{y,6}$	99.1%	98.8%	0.016		$v_{y,5}$	98.4%	98.4%	0.033
	$v_{x,9}$	96.8%	93.9%	0.024		$v_{x,6}$	90.2%	88.7%	0.039
	$v_{y,9}$	98.8%	98.3%	0.019		$v_{y,6}$	97.4%	96.6%	0.045
ANN_89	$v_{x,8}$	97.6%	95.8%	0.013		$v_{x,8}$	89.6%	88.1%	0.045
	$v_{y,8}$	99.6%	99.3%	0.015		$v_{y,8}$	98.1%	97.8%	0.038
	$v_{x,9}$	97.5%	95.0%	0.017		$v_{x,9}$	85.2%	82.3%	0.058
	$v_{y,9}$	99.2%	98.4%	0.020		$v_{y,9}$	96.9%	96.2%	0.053

## Data Availability

The original contributions presented in the study are included in the article; further inquires can be directed to the corresponding author.
